# Solitary CNS Metastasis on Initial Presentation of High Grade Serous Carcinoma of the Fallopian Tube

**DOI:** 10.1155/2018/6930986

**Published:** 2018-12-05

**Authors:** Felicity Harl, Cassandra Niemi, Lori Mankowski Gettle, Paul Weisman, Stephen Rose

**Affiliations:** ^1^University of Wisconsin School of Medicine and Public Health, Madison, WI, USA; ^2^Compass Oncology, Portland, OR, USA; ^3^Department of Radiology, University of Wisconsin School of Medicine and Public Health, Madison, WI, USA; ^4^Department of Pathology and Laboratory Medicine, University of Wisconsin School of Medicine and Public Health, Madison, WI, USA; ^5^Department of Obstetrics and Gynecology, University of Wisconsin School of Medicine and Public Health, Madison, WI, USA

## Abstract

A 68-year-old woman presented with a three-week history of confusion and anomic aphasia. Imaging of her head demonstrated a single large left frontal mass. Pathology revealed metastatic adenocarcinoma of Müllerian origin. Subsequent surgery revealed a small primary site in a fallopian tube, high left para-aortic lymphadenopathy, and no disseminated intraperitoneal disease. This case was remarkable in that CNS metastasis was her presenting symptom and was restricted to a solitary brain lesion, and other disease sites were limited to retroperitoneal lymphadenopathy and a small fallopian tube primary.

## 1. Introduction

In the past decade, advances in histopathology and genetics have resulted in a paradigm shift wherein ovarian, fallopian tube, and peritoneal carcinomas are considered a spectrum of disease originating from the Müllerian compartment rather than separate entities [1]. Specifically, the fimbrial fallopian tube epithelium is thought to represent the site of origin of these tumors [2]. Subsequently, FIGO staging for these cancers was revised to include all of them under the umbrella of ovarian cancer staging classification in 2013 [3]. Though this case is specifically about an instance of a primary high grade serous carcinoma of the fallopian tube, this case report adds to the body of literature about all adenocarcinomas of Müllerian origin—ovarian, fallopian tube, and peritoneal.

Ovarian cancer is the second most common gynecological cancer [4]. In 2015, 21,429 women were diagnosed with ovarian cancer and 13,920 women died from the disease [4]. Effective screening methods do not exist and early signs are vague and nonspecific; therefore, these cancers are usually identified at more advanced stages when metastases are already present [5]. Prognosis is poor for advanced stage disease with a less than 40% survival rate at five years [5].

Common sites of distant metastases include the liver, pleura, and lungs [6]. Metastases to the central nervous system are less common and are usually a late manifestation [7]. Cormio [6] found no brain metastases present at the time of diagnosis in a study of 162 patients diagnosed with primary ovarian carcinoma. We present a case of high grade serous carcinoma of the fallopian tube in which the initial presentation was that of neurological symptoms stemming from a solitary brain mass in the left frontal lobe. Upon total laparoscopic hysterectomy and bilateral salpingo-oophorectomy, evidence of a small fallopian tube primary was found. At the time, there was lymphadenopathy behind and above the left renal vein but no other evidence of metastases in the chest, abdomen, and pelvis. There are only a handful of reports on cases in which CNS metastases were present on initial presentation of fallopian tube and ovarian cancers before or at the time of diagnosis of the primary cancer [8–11]. This case is further noteworthy because the brain lesion was solitary, while the fallopian tube primary site was not seen on initial imaging and subsequently found to be relatively small.

## 2. Case Presentation

**Figure 1:**
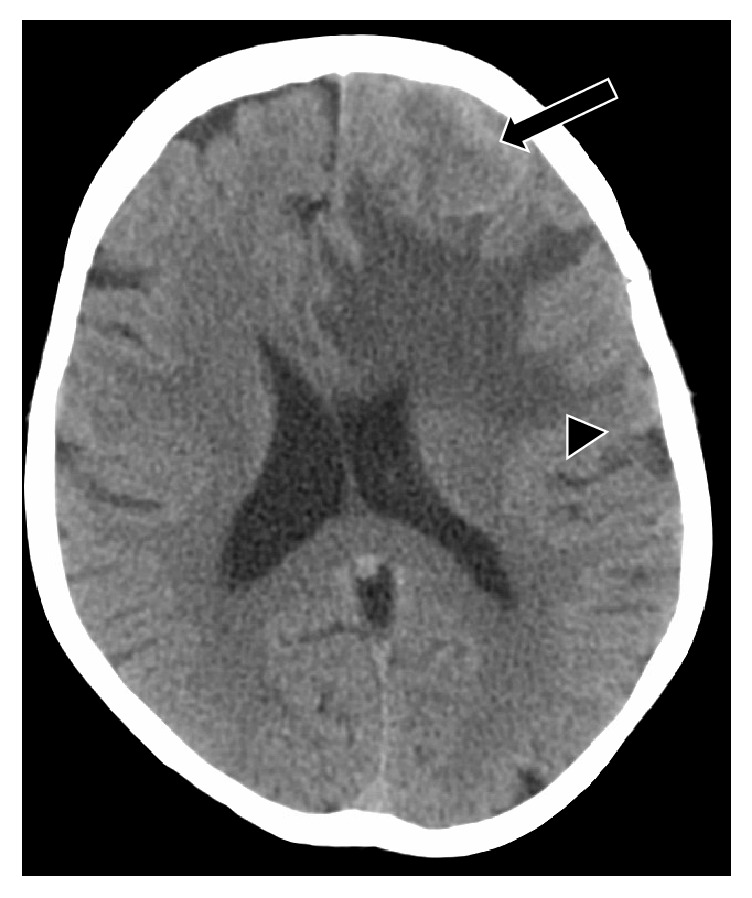
At initial presentation with altered mental status, CT head without contrast showed a left frontal mass (arrow) with surrounding edema (arrow head) and midline shift.

**Figure 2:**
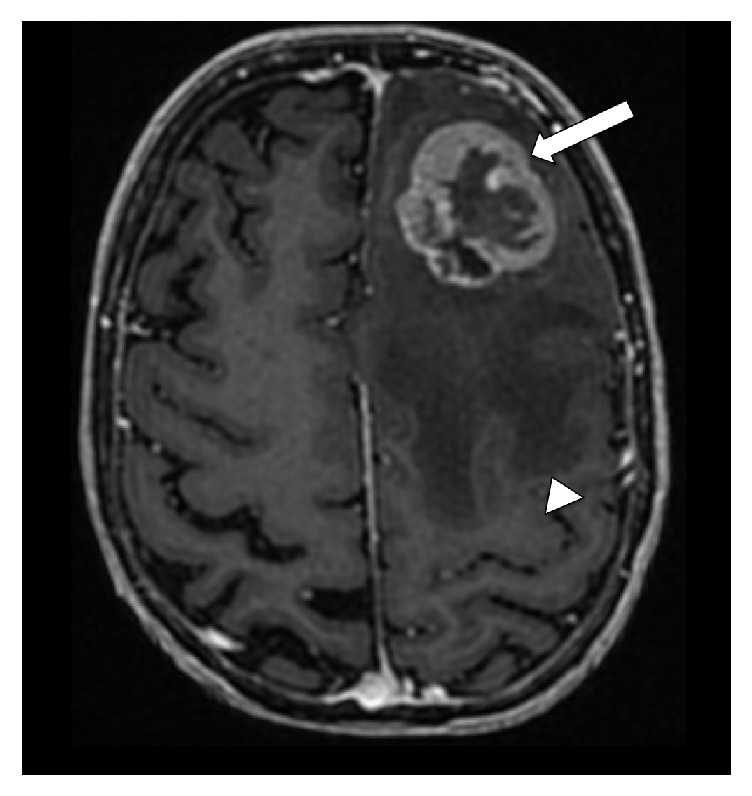
Surgical planning MRI with contrast shows an irregular left frontal mass (arrow) with surrounding edema (arrow head).

**Figure 3:**
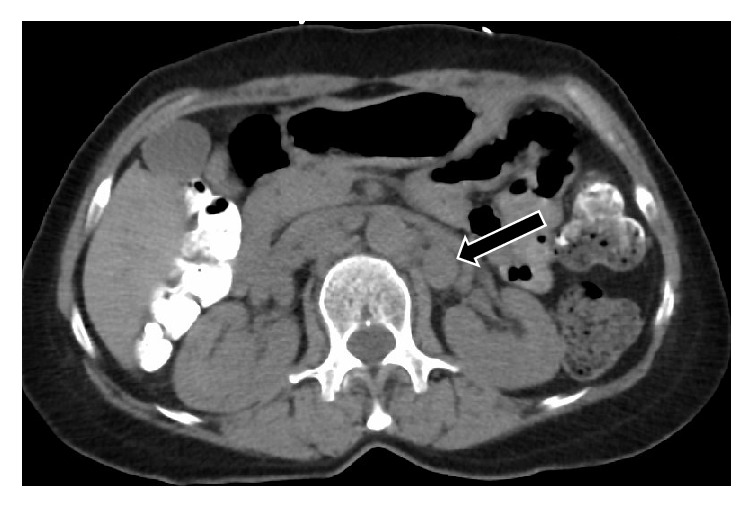
CT of the chest, abdomen, and pelvis performed without contrast (due to allergy) to evaluate for primary malignancy. The only finding was retroperitoneal lymphadenopathy notably of the left para-aortic nodes posterior to the renal vein (arrow).

**Figure 4:**
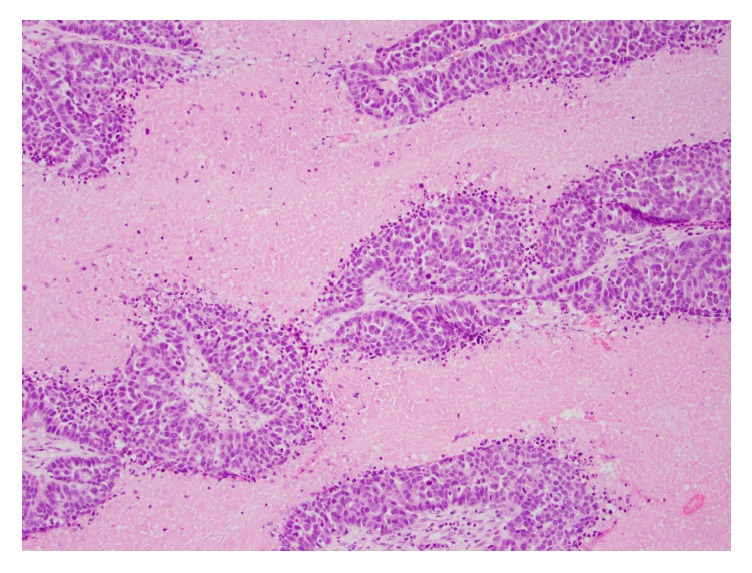
Tumor involving the brain. High grade serous carcinoma in the brain, morphologically resembling the tumor seen in the fallopian tube (hematoxylin and eosin, 100x magnification).

**Figure 5:**
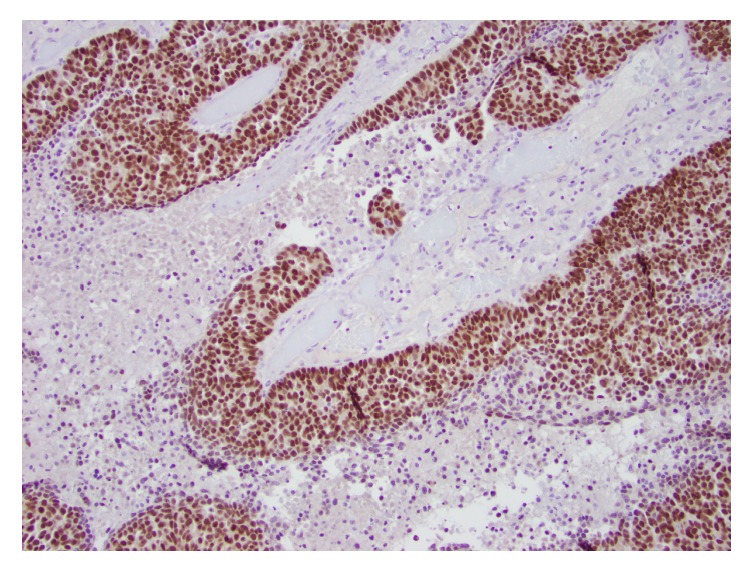
Tumor involving the brain. PAX8 immunohistochemical stain shows similarly strong nuclear staining in the tumor (100x magnification).

A 68-year-old woman presented with a three-week history of mild confusion and anomic aphasia. Her past medical history was significant for remote papillary thyroid cancer and scalp radiation for ringworm. A noncontrast CT of the head found extensive left frontoparietal vasogenic edema with mass effect of the underlying brain parenchyma and a left frontal mass-like density (Figure 1). An MRI of the head demonstrated a large mass in the left frontal lobe with thick enhancing borders and a 9-mm left-to-right midline shift (Figure 2). A CT of the chest, abdomen, and pelvis performed that day showed lymphadenopathy behind and above the left renal vein with the dominant node measuring 1.7 cm (Figure 3). The patient underwent a left frontal craniotomy and microsurgical tumor removal. Pathology of the specimen revealed a large metastatic carcinoma of Müllerian origin with enlarged nuclei and nucleoli, abundant mitosis, and intraluminal necrosis (Figures 4 and 5). The tumor was positive for PAX-8 and CK7 by immunohistochemistry.

**Figure 6:**
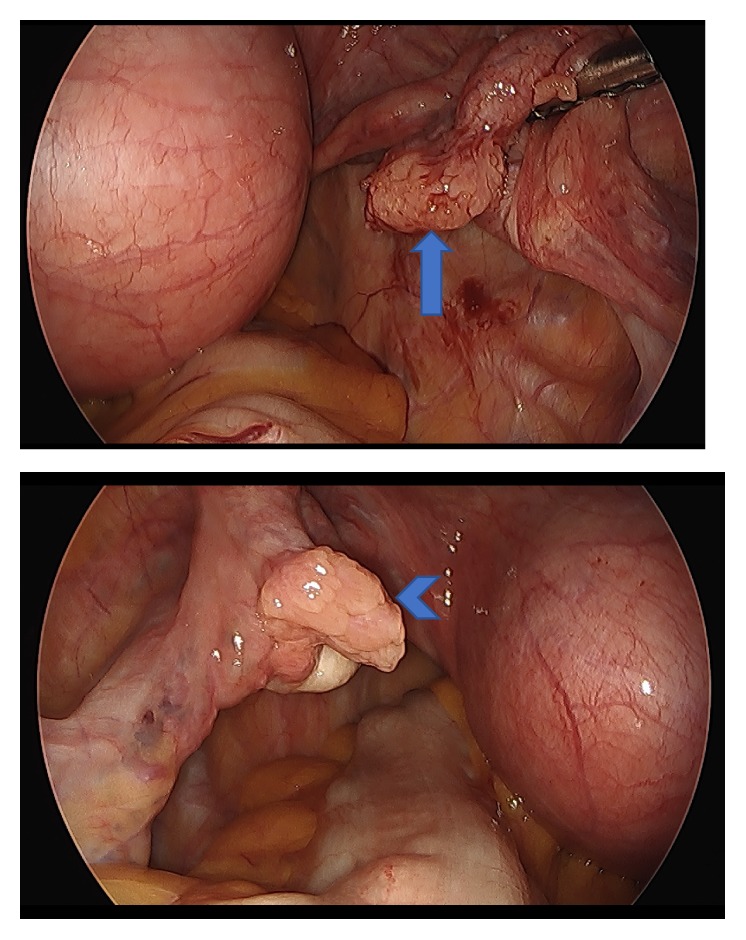
Images from laparoscopic total hysterectomy and bilateral salpingo-oophorectomy. Right fallopian tube fimbria with darker appearance (arrow). Normal appearance of left fallopian tube (arrow head).

**Figure 7:**
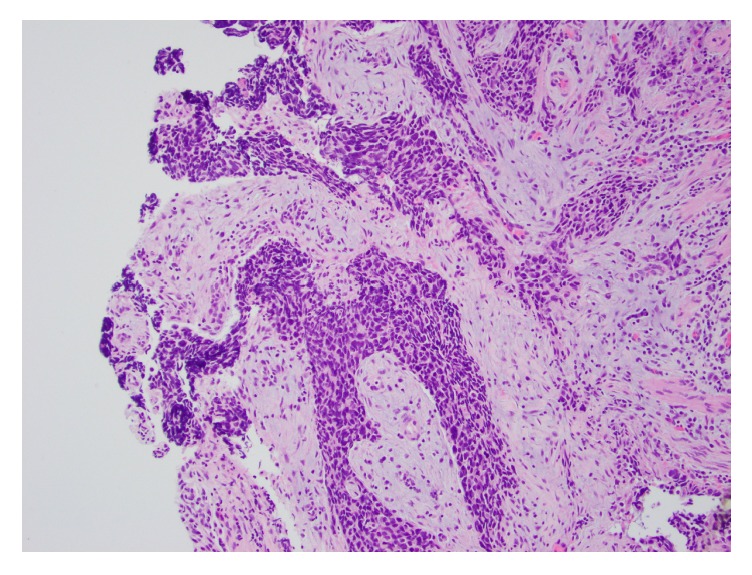
Tumor involving the fallopian tube. High grade serous carcinoma at the surface of the fallopian tube (hematoxylin and eosin, 100x magnification).

**Figure 8:**
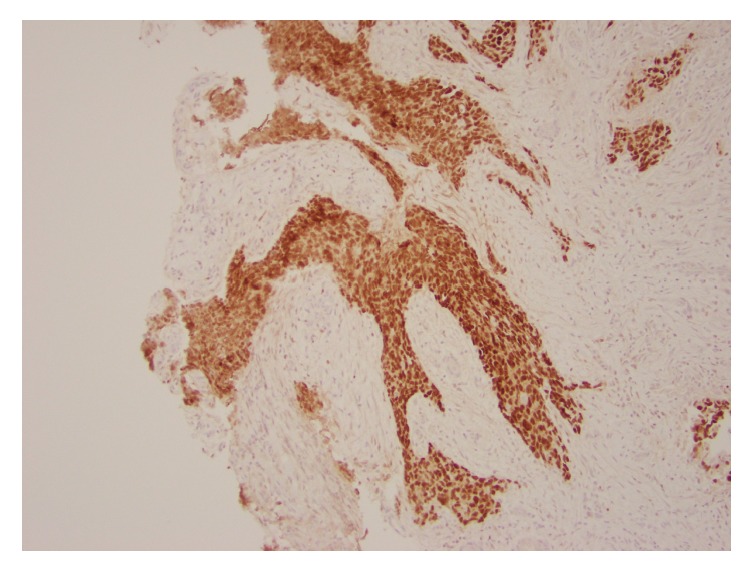
Tumor involving the fallopian tube. PAX8 immunohistochemical stain shows strong nuclear staining in the tumor (100x magnification).

One month later, the patient underwent a total laparoscopic hysterectomy, bilateral salpingo-oophorectomy, and cystoscopy. Intraoperative findings were notable for a darkened area on the right fallopian tube fimbria which was concerning for malignancy. The abdominal survey was otherwise normal (Figure 6). Final pathology revealed high grade serous carcinoma identified only at the fimbrial end of the right fallopian tube, measuring 0.6 cm (Figures 7 and 8). By immunohistochemistry, the tumor was positive for p53, PAX-8, and WT-1 and negative for TTF-1.

The patient underwent fractionated stereotactic radiotherapy to the left frontal resection cavity. Patient received a cumulative dose of 30 Gy in 5 fractions. After completing radiotherapy, she began adjuvant chemotherapy with 6 cycles of carboplatin AUC of 6 every 21 days and paclitaxel 80 mg/m2 weekly. She developed grade 3 neutropenia and was switched to carboplatin AUC of 5 and paclitaxel 150 mg/m2 every 21 days for cycles 5 and 6. She later received genetic testing using a 19-gene breast and ovarian cancer panel which was negative for mutations. She had a complete response to adjuvant treatment with a CA 125 of 22 and negative imaging.

## 3. Discussion

CNS involvement is rare in carcinomas of Müllerian origin, with an estimated incidence of around 5% in ovarian cancer [12]. Intraperitoneal dissemination is the most common route of metastatic spread of these cancers [6]. Cormio [6] found that only 22% of the patients ever developed distant metastasis, defined as stage IV disease per FIGO classification, during the course of ovarian carcinoma.

CNS involvement as the initial presentation of disease is even more rare [6] with only a handful of case reports in the English literature [8–11]. Bakar and Tekkök [8] described a case of undifferentiated ovarian carcinoma in a patient whose presenting symptoms were headache and vision changes, for which subsequent imaging demonstrated multiple ring-enhancing intracranial tumors. Mastsunami [9] reported a case of poorly differentiated adenocarcinoma (endometrioid type) of ovarian origin in a woman whose presenting symptoms were progressive nausea, fatigue, and headache due to a solitary intracranial tumor. Alafaci [10] reported a case of ovarian papillary serous carcinoma in a patient who presented with headache, vertigo, and visual disturbances due to a solitary parietal lesion. Finally, Raff [11] presented a case of a malignant fallopian tube carcinoma in a patient with a three-week history of left-sided weakness and a solitary parietal lesion. These four cases mirror our patient's case in that they all involve carcinomas of Müllerian origin in patients whose presenting symptoms were due to CNS metastasis, three of which were solitary cerebral lesions. Our case report underscores this potentially underrecognized pattern of early CNS metastasis from adenocarcinomas of Müllerian origin.

The location of CNS lesions has significant implications on presentation of symptoms. Brain metastases are most common in the cerebral hemispheres with parietal, frontal, and temporal lobes as the most frequent sites (in that order) [12, 13]. This aligns with the location of our patient's single metastatic lesion in the frontal lobe. Stanislawiak, Lubin, and Markowsa [13] reported headache as the most common presentation of CNS metastasis, which they attributed to increased intracranial pressure. By contrast, our patient presented with mild confusion and anosmia.

The prognosis of Müllerian adenocarcinomas is poor in general with only 40% of women surviving to five years after diagnosis and most patients dying within one year of diagnosis of distant metastases [5, 6]. Patients with CNS disease fare even worse. A study of 50 patients with metastatic ovarian cancer, 7 of whom had brain metastases, found that patients with brain metastases have a median survival of only 10 months [6]. However, the number of lesions appears to have prognostic significance: One study showed that patients with solitary brain lesions had a better prognosis than those with multiple lesions. Patients with multiple lesions had a fourfold greater chance of dying after diagnosis (HR 4.4, 95% CI, 2.0-9.7,* P*<0.001) [14].

The incidence of CNS metastases from primary ovarian carcinomas appears to be increasing [15]. Possible reasons for this include increased accessibility of imaging (earlier and more sensitive diagnosis) and prolonged survival related to more aggressive treatment [15]. In addition, it has been suggested that chemotherapy may compromise the blood brain barrier, increasing the susceptibility to CNS metastases later in the course of the disease [13]. However, this effect cannot account for CNS metastasis as the primary presentation, as in our patient. Further studies are necessary in order to identify the factors, both host and tumor based, that could make the CNS prone to metastasis so early in the disease process.

## 4. Conclusion 

This patient's fallopian tube carcinoma is remarkable for the small size of the occult primary tumor and the early metastatic spread to the CNS (as a solitary lesion), which was ultimately responsible for the patient's presenting symptoms. This case underscores the importance of considering the brain as a potential site of metastasis, even early in the disease course of Müllerian adenocarcinomas, contrary to the dogma that CNS involvement is a late finding. Finally, based on the limited data on CNS involvement by Müllerian adenocarcinoma, there is some evidence that patients with only solitary brain metastases may have an improved prognosis.
